# 3-[(4-Chloro­phen­yl)sulfin­yl]-2,4,6,7-tetra­methyl-1-benzofuran

**DOI:** 10.1107/S1600536810042339

**Published:** 2010-11-06

**Authors:** Hong Dae Choi, Pil Ja Seo, Byeng Wha Son, Uk Lee

**Affiliations:** a Department of Chemistry, Dongeui University, San 24 Kaya-dong Busanjin-gu, Busan 614-714, Republic of Korea; b Department of Chemistry, Pukyong National University, 599-1 Daeyeon 3-dong Nam-gu, Busan 608-737, Republic of Korea

## Abstract

In the title compound, C_18_H_17_ClO_2_S, the 4-chloro­phenyl ring is oriented approximately perpendicular to the mean plane of the benzofuran ring [dihedral angle = 87.49 (5)°]. In the crystal, mol­ecules are linked through weak inter­molecular C—H⋯π inter­actions, forming left- and right-handed pseudo-helices along the *a* axis.

## Related literature

For the biological activity of benzofuran compounds, see: Aslam *et al.* (2006 ▶); Galal *et al.* (2009 ▶); Khan *et al.* (2005 ▶). For natural products with benzofuran rings, see: Akgul & Anil (2003 ▶); Soekamto *et al.* (2003 ▶). For the structures of related 3-(4–chloro­phenyl­sulfin­yl)-2-methyl-1-benzofuran derivatives, see: Choi *et al.* (2010**a* ▶,*b* ▶,c* ▶).
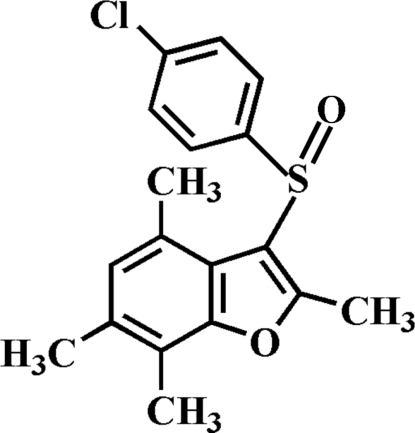


## Experimental

### 

#### Crystal data

C_18_H_17_ClO_2_S
*M* *_r_* = 332.83
Orthorhombic, 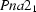

*a* = 12.2329 (4) Å
*b* = 20.1499 (7) Å
*c* = 6.4840 (2) Å
*V* = 1598.25 (9) Å^3^
*Z* = 4
Mo *K*α radiation
μ = 0.37 mm^−1^
*T* = 173 K
0.33 × 0.16 × 0.10 mm

#### Data collection

Bruker SMART APEXII CCD diffractometer
Absorption correction: multi-scan (*SADABS*; Bruker, 2009 ▶) *T*_min_ = 0.651, *T*_max_ = 0.746
8651 measured reflections
3392 independent reflections
2809 reflections with *I* > 2σ(*I*)
*R*_int_ = 0.034

#### Refinement

*R*[*F* ^2^ > 2σ(*F* ^2^)] = 0.038
*wR*(*F* ^2^) = 0.090
*S* = 1.05
3392 reflections
203 parameters
1 restraint
H-atom parameters constrained
Δρ_max_ = 0.20 e Å^−3^
Δρ_min_ = −0.25 e Å^−3^
Absolute structure: Flack (1983 ▶), 1382 Friedel pairs
Flack parameter: −0.03 (6)

### 

Data collection: *APEX2* (Bruker, 2009 ▶); cell refinement: *SAINT* (Bruker, 2009 ▶); data reduction: *SAINT*; program(s) used to solve structure: *SHELXS97* (Sheldrick, 2008 ▶); program(s) used to refine structure: *SHELXL97* (Sheldrick, 2008 ▶); molecular graphics: *ORTEP-3* (Farrugia, 1997 ▶) and *DIAMOND* (Brandenburg, 1998 ▶); software used to prepare material for publication: *SHELXL97*.

## Supplementary Material

Crystal structure: contains datablocks global, I. DOI: 10.1107/S1600536810042339/bh2316sup1.cif

Structure factors: contains datablocks I. DOI: 10.1107/S1600536810042339/bh2316Isup2.hkl

Additional supplementary materials:  crystallographic information; 3D view; checkCIF report

## Figures and Tables

**Table 1:** Hydrogen-bond geometry (Å, °) *Cg*1 and *Cg*2 are the centroids of the C13–C18 4-chloro­phenyl ring and the C2–C7 benzene ring, respectively.

*D*—H⋯*A*	*D*—H	H⋯*A*	*D*⋯*A*	*D*—H⋯*A*
C11—H11*A*⋯*Cg*1^i^	0.96	2.76	3.613 (3)	148
C12—H12*B*⋯*Cg*2^ii^	0.96	2.83	3.653 (3)	144

Symmetry codes: (i) 
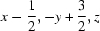
; (ii) 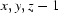.

**Fig. 1.**
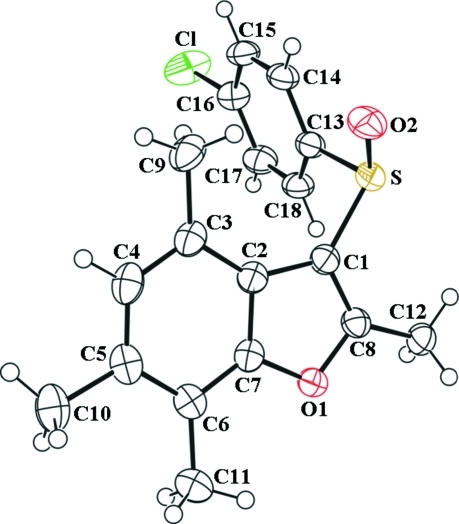
The molecular structure of the title compound with the atom numbering scheme. Displacement ellipsoids are drawn at the 50% probability level. H atoms are presented as small spheres of arbitrary radius.

**Fig. 2.**
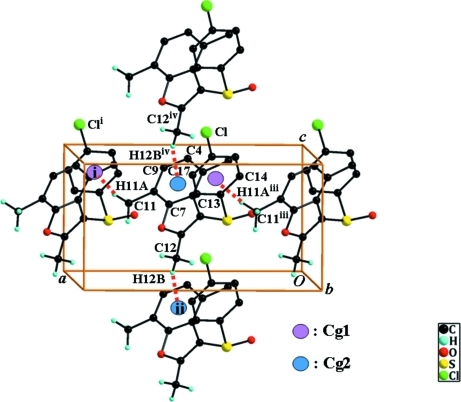
A view of the C—H···π interactions (dotted lines) in the crystal structure of the title compound. Cg1 and Cg2 denote the centroids of the C13···C18 4-chlorophenyl ring and the C2···C7 benzene ring, respectively. [Symmetry codes: (i) x-1/2, -y+3/2, z; (ii) x, y, z-1; (iii) x-1/2, -y+3/2, z; (iv) x, y, z+1].

## supplementary crystallographic information

### Comment

A series of benzofuran ring system have received much attention in view of their interesting pharmacological properties such as antifungal, antimicrobial, antitumor and antiviral activities (Aslam *et al.*, 2006; Galal *et al.*, 2009; Khan *et al.*, 2005). These compounds widely occur in nature (Akgul & Anil, 2003; Soekamto *et al.*, 2003). As a part of our study of the substituent effect on the solid state structures of 3-(4-chlorophenylsulfinyl)-2-methyl-1-benzofuran analogues (Choi *et al.*, 2010**a*,*b*,c*), we report herein on the crystal structure of the title compound.

In the title molecule (Fig. 1), the benzofuran unit is essentially planar, with a mean deviation of 0.011 (2) Å from the least-squares plane defined by the nine constituent atoms. The 4-chlorophenyl ring makes a dihedral angle of 87.49 (5)° with the mean plane of the benzofuran fragment. The crystal packing (Fig. 2) is stabilized by weak intermolecular C—H···π interactions; the first one between a methyl H atom and the 4-chlorophenyl ring (C11—H11A···Cg1^i^; Table 1, Cg1 is the centroid of the C13···C18 4–chlorophenyl ring), and the second one between a methyl H atom and the benzene ring (C12—H12B···Cg2^ii^; Table 1, Cg2 is the centroid of the C2···C7 benzene ring). The title compound crystallizes in the non-centrosymmetric space group *Pna*2_1_ in spite of having no asymmetric C atoms. The space group is caused by a right-hand pseudo-helix along the *a* axis (Fig. 2).

### Experimental

77% 3-chloroperoxybenzoic acid (269 mg, 1.2 mmol) was added in small portions to a stirred solution of 3-(4-chlorophenylsulfanyl)-2,4,6,7-tetramethyl-1-benzofuran (348 mg, 1.0 mmol) in dichloromethane (40 mL) at 273 K. After being stirred at room temperature for 4h, the mixture was washed with saturated sodium bicarbonate solution and the organic layer was separated, dried over magnesium sulfate, filtered and concentrated at reduced pressure. The residue was purified by column chromatography (hexane-ethyl acetate, 2:1 *v*/*v*) to afford the title compound as a colorless solid [yield 76%, m.p. 442–443 K; *R*_f_ = 0.67 (hexane-ethyl acetate, 2:1 *v*/*v*)]. Single crystals suitable for X–ray diffraction were prepared by slow evaporation of a solution of the title compound in ethyl acetate at room temperature.

### Refinement

All H atoms were positioned geometrically and refined using a riding model, with C—H = 0.93 Å for aryl and 0.96 Å for methyl H atoms. *U*_iso_(H) = 1.2*U*_eq_(C) for aryl and 1.5*U*_eq_(C) for methyl H atoms.

### Crystal data

**Table:**

C_18_H_17_ClO_2_S	*D*_x_ = 1.383 Mg m^−^^3^
*M**_r_* = 332.83	Melting point: 442 K
Orthorhombic, *P**n**a*2_1_	Mo *K*α radiation, λ = 0.71073 Å
Hall symbol: P 2c -2n	Cell parameters from 2945 reflections
*a* = 12.2329 (4) Å	θ = 2.6–26.8°
*b* = 20.1499 (7) Å	µ = 0.37 mm^−^^1^
*c* = 6.4840 (2) Å	*T* = 173 K
*V* = 1598.25 (9) Å^3^	Block, colourless
*Z* = 4	0.33 × 0.16 × 0.10 mm
*F*(000) = 696	

### Data collection

**Table:**

Bruker SMART APEXII CCD diffractometer	3392 independent reflections
Radiation source: rotating anode	2809 reflections with *I* > 2σ(*I*)
graphite multilayer	*R*_int_ = 0.034
Detector resolution: 10.0 pixels mm^-1^	θ_max_ = 27.6°, θ_min_ = 2.0°
φ and ω scans	*h* = −15→11
Absorption correction: multi-scan (*SADABS*; Bruker, 2009)	*k* = −26→20
*T*_min_ = 0.651, *T*_max_ = 0.746	*l* = −8→8
8651 measured reflections	

### Refinement

**Table:**

Refinement on *F*^2^	Secondary atom site location: difference Fourier map
Least-squares matrix: full	Hydrogen site location: difference Fourier map
*R*[*F*^2^ > 2σ(*F*^2^)] = 0.038	H-atom parameters constrained
*wR*(*F*^2^) = 0.090	*w* = 1/[σ^2^(*F*_o_^2^) + (0.0426*P*)^2^ + 0.1096*P*] where *P* = (*F*_o_^2^ + 2*F*_c_^2^)/3
*S* = 1.05	(Δ/σ)_max_ < 0.001
3392 reflections	Δρ_max_ = 0.20 e Å^−^^3^
203 parameters	Δρ_min_ = −0.25 e Å^−^^3^
1 restraint	Absolute structure: Flack (1983), 1382 Friedel pairs
0 constraints	Flack parameter: −0.03 (6)
Primary atom site location: structure-invariant direct methods	

### Fractional atomic coordinates and isotropic or equivalent isotropic displacement parameters (Å^2^)

**Table:**

	*x*	*y*	*z*	*U*_iso_*/*U*_eq_	
Cl	0.48480 (5)	0.93993 (4)	1.24227 (15)	0.0644 (2)	
S	0.37852 (4)	0.74513 (3)	0.51507 (13)	0.03587 (15)	
O1	0.64405 (11)	0.64000 (7)	0.4393 (2)	0.0317 (4)	
O2	0.27233 (11)	0.71240 (9)	0.5638 (3)	0.0462 (5)	
C1	0.48689 (15)	0.68792 (10)	0.5388 (4)	0.0304 (5)	
C2	0.51433 (16)	0.63782 (11)	0.6943 (4)	0.0308 (5)	
C3	0.47011 (16)	0.61312 (12)	0.8785 (4)	0.0350 (5)	
C4	0.52973 (19)	0.56352 (12)	0.9760 (4)	0.0394 (6)	
H4	0.5030	0.5469	1.1000	0.047*	
C5	0.62780 (17)	0.53683 (12)	0.8996 (4)	0.0368 (6)	
C6	0.67069 (16)	0.55999 (10)	0.7143 (4)	0.0331 (5)	
C7	0.61165 (16)	0.61008 (11)	0.6216 (4)	0.0301 (5)	
C8	0.56685 (15)	0.68753 (11)	0.3949 (4)	0.0312 (5)	
C9	0.36522 (17)	0.63876 (14)	0.9713 (4)	0.0443 (7)	
H9A	0.3772	0.6823	1.0272	0.066*	
H9B	0.3418	0.6094	1.0791	0.066*	
H9C	0.3099	0.6409	0.8665	0.066*	
C10	0.6861 (2)	0.48422 (12)	1.0225 (5)	0.0506 (7)	
H10A	0.7622	0.4953	1.0329	0.076*	
H10B	0.6783	0.4421	0.9548	0.076*	
H10C	0.6549	0.4817	1.1581	0.076*	
C11	0.77427 (18)	0.53408 (12)	0.6181 (4)	0.0423 (6)	
H11A	0.8362	0.5515	0.6912	0.063*	
H11B	0.7777	0.5477	0.4764	0.063*	
H11C	0.7750	0.4865	0.6255	0.063*	
C12	0.58942 (18)	0.72823 (11)	0.2117 (4)	0.0370 (5)	
H12A	0.6521	0.7557	0.2370	0.055*	
H12B	0.5273	0.7557	0.1824	0.055*	
H12C	0.6036	0.6998	0.0959	0.055*	
C13	0.41185 (16)	0.79762 (10)	0.7289 (4)	0.0322 (5)	
C14	0.33260 (16)	0.81193 (11)	0.8759 (4)	0.0353 (5)	
H14	0.2642	0.7920	0.8672	0.042*	
C15	0.35484 (16)	0.85543 (11)	1.0343 (5)	0.0361 (5)	
H15	0.3025	0.8644	1.1346	0.043*	
C16	0.45579 (17)	0.88545 (11)	1.0414 (4)	0.0381 (6)	
C17	0.53552 (17)	0.87296 (12)	0.8939 (4)	0.0405 (6)	
H17	0.6029	0.8942	0.9008	0.049*	
C18	0.51360 (16)	0.82887 (11)	0.7378 (4)	0.0367 (6)	
H18	0.5663	0.8199	0.6384	0.044*	

### Atomic displacement parameters (Å^2^)

**Table:**

	*U*^11^	*U*^22^	*U*^33^	*U*^12^	*U*^13^	*U*^23^
Cl	0.0533 (4)	0.0701 (5)	0.0699 (5)	−0.0190 (3)	0.0152 (4)	−0.0291 (4)
S	0.0257 (2)	0.0485 (3)	0.0334 (3)	0.0036 (2)	−0.0013 (3)	0.0026 (3)
O1	0.0293 (6)	0.0334 (8)	0.0324 (9)	0.0032 (6)	0.0014 (7)	0.0023 (7)
O2	0.0239 (7)	0.0632 (11)	0.0516 (12)	−0.0027 (7)	−0.0036 (8)	−0.0053 (9)
C1	0.0256 (9)	0.0357 (12)	0.0299 (12)	−0.0028 (8)	−0.0019 (11)	−0.0005 (11)
C2	0.0251 (9)	0.0363 (13)	0.0310 (13)	−0.0064 (8)	−0.0011 (10)	0.0004 (10)
C3	0.0307 (10)	0.0434 (14)	0.0310 (13)	−0.0115 (10)	0.0007 (11)	0.0016 (11)
C4	0.0425 (12)	0.0428 (14)	0.0330 (15)	−0.0160 (10)	−0.0024 (11)	0.0065 (11)
C5	0.0388 (11)	0.0315 (13)	0.0401 (14)	−0.0094 (9)	−0.0084 (12)	0.0049 (12)
C6	0.0318 (10)	0.0291 (12)	0.0384 (14)	−0.0052 (9)	−0.0054 (11)	0.0002 (11)
C7	0.0288 (10)	0.0324 (12)	0.0292 (13)	−0.0075 (9)	−0.0007 (10)	0.0017 (10)
C8	0.0268 (10)	0.0343 (12)	0.0324 (12)	−0.0009 (9)	−0.0042 (11)	−0.0024 (10)
C9	0.0385 (11)	0.0578 (16)	0.0366 (17)	−0.0109 (11)	0.0089 (12)	0.0031 (12)
C10	0.0594 (14)	0.0451 (15)	0.0472 (15)	−0.0088 (12)	−0.0102 (16)	0.0129 (14)
C11	0.0396 (12)	0.0361 (13)	0.0513 (17)	0.0052 (10)	−0.0004 (13)	0.0006 (12)
C12	0.0341 (11)	0.0457 (14)	0.0312 (13)	−0.0004 (10)	0.0025 (12)	0.0069 (12)
C13	0.0268 (9)	0.0334 (12)	0.0363 (13)	0.0047 (9)	0.0039 (11)	0.0074 (12)
C14	0.0238 (9)	0.0403 (13)	0.0419 (14)	0.0029 (9)	0.0036 (12)	0.0044 (12)
C15	0.0297 (10)	0.0376 (12)	0.0410 (15)	0.0037 (9)	0.0109 (13)	0.0020 (13)
C16	0.0355 (10)	0.0360 (13)	0.0426 (15)	−0.0007 (9)	0.0038 (12)	−0.0022 (12)
C17	0.0282 (10)	0.0423 (14)	0.0510 (16)	−0.0048 (10)	0.0047 (12)	0.0017 (13)
C18	0.0275 (10)	0.0405 (13)	0.0421 (14)	0.0035 (9)	0.0086 (12)	0.0070 (13)

### Geometric parameters (Å, °)

**Table:**

Cl—C16	1.740 (3)	C9—H9C	0.9600
S—O2	1.4907 (15)	C10—H10A	0.9600
S—C1	1.763 (2)	C10—H10B	0.9600
S—C13	1.791 (3)	C10—H10C	0.9600
O1—C8	1.375 (2)	C11—H11A	0.9600
O1—C7	1.385 (3)	C11—H11B	0.9600
C1—C8	1.352 (3)	C11—H11C	0.9600
C1—C2	1.466 (3)	C12—H12A	0.9600
C2—C7	1.397 (3)	C12—H12B	0.9600
C2—C3	1.402 (3)	C12—H12C	0.9600
C3—C4	1.389 (3)	C13—C14	1.390 (3)
C3—C9	1.508 (3)	C13—C18	1.396 (3)
C4—C5	1.405 (3)	C14—C15	1.377 (4)
C4—H4	0.9300	C14—H14	0.9300
C5—C6	1.391 (4)	C15—C16	1.376 (3)
C5—C10	1.506 (3)	C15—H15	0.9300
C6—C7	1.379 (3)	C16—C17	1.389 (3)
C6—C11	1.506 (3)	C17—C18	1.373 (4)
C8—C12	1.470 (3)	C17—H17	0.9300
C9—H9A	0.9600	C18—H18	0.9300
C9—H9B	0.9600		
			
O2—S—C1	110.33 (10)	C5—C10—H10B	109.5
O2—S—C13	107.20 (11)	H10A—C10—H10B	109.5
C1—S—C13	98.48 (10)	C5—C10—H10C	109.5
C8—O1—C7	106.57 (16)	H10A—C10—H10C	109.5
C8—C1—C2	107.76 (18)	H10B—C10—H10C	109.5
C8—C1—S	119.19 (18)	C6—C11—H11A	109.5
C2—C1—S	133.01 (17)	C6—C11—H11B	109.5
C7—C2—C3	118.3 (2)	H11A—C11—H11B	109.5
C7—C2—C1	103.78 (19)	C6—C11—H11C	109.5
C3—C2—C1	137.9 (2)	H11A—C11—H11C	109.5
C4—C3—C2	116.1 (2)	H11B—C11—H11C	109.5
C4—C3—C9	120.8 (2)	C8—C12—H12A	109.5
C2—C3—C9	123.1 (2)	C8—C12—H12B	109.5
C3—C4—C5	124.3 (2)	H12A—C12—H12B	109.5
C3—C4—H4	117.9	C8—C12—H12C	109.5
C5—C4—H4	117.9	H12A—C12—H12C	109.5
C6—C5—C4	119.9 (2)	H12B—C12—H12C	109.5
C6—C5—C10	121.0 (2)	C14—C13—C18	120.0 (2)
C4—C5—C10	119.1 (2)	C14—C13—S	119.65 (17)
C7—C6—C5	115.1 (2)	C18—C13—S	120.10 (19)
C7—C6—C11	120.9 (2)	C15—C14—C13	120.4 (2)
C5—C6—C11	124.0 (2)	C15—C14—H14	119.8
C6—C7—O1	122.7 (2)	C13—C14—H14	119.8
C6—C7—C2	126.3 (2)	C16—C15—C14	118.8 (2)
O1—C7—C2	110.98 (19)	C16—C15—H15	120.6
C1—C8—O1	110.9 (2)	C14—C15—H15	120.6
C1—C8—C12	133.7 (2)	C15—C16—C17	121.8 (2)
O1—C8—C12	115.38 (18)	C15—C16—Cl	119.1 (2)
C3—C9—H9A	109.5	C17—C16—Cl	119.11 (17)
C3—C9—H9B	109.5	C18—C17—C16	119.2 (2)
H9A—C9—H9B	109.5	C18—C17—H17	120.4
C3—C9—H9C	109.5	C16—C17—H17	120.4
H9A—C9—H9C	109.5	C17—C18—C13	119.8 (2)
H9B—C9—H9C	109.5	C17—C18—H18	120.1
C5—C10—H10A	109.5	C13—C18—H18	120.1
			
O2—S—C1—C8	137.63 (18)	C8—O1—C7—C2	0.1 (2)
C13—S—C1—C8	−110.40 (19)	C3—C2—C7—C6	−0.4 (3)
O2—S—C1—C2	−44.6 (3)	C1—C2—C7—C6	−179.7 (2)
C13—S—C1—C2	67.3 (2)	C3—C2—C7—O1	−179.92 (18)
C8—C1—C2—C7	−1.4 (2)	C1—C2—C7—O1	0.7 (2)
S—C1—C2—C7	−179.29 (18)	C2—C1—C8—O1	1.5 (2)
C8—C1—C2—C3	179.5 (3)	S—C1—C8—O1	179.78 (14)
S—C1—C2—C3	1.6 (4)	C2—C1—C8—C12	−175.5 (2)
C7—C2—C3—C4	1.6 (3)	S—C1—C8—C12	2.8 (4)
C1—C2—C3—C4	−179.4 (2)	C7—O1—C8—C1	−1.1 (2)
C7—C2—C3—C9	−179.6 (2)	C7—O1—C8—C12	176.55 (19)
C1—C2—C3—C9	−0.5 (4)	O2—S—C13—C14	−13.4 (2)
C2—C3—C4—C5	−1.3 (4)	C1—S—C13—C14	−127.83 (19)
C9—C3—C4—C5	179.8 (2)	O2—S—C13—C18	172.49 (18)
C3—C4—C5—C6	−0.2 (4)	C1—S—C13—C18	58.0 (2)
C3—C4—C5—C10	178.6 (2)	C18—C13—C14—C15	−1.9 (3)
C4—C5—C6—C7	1.4 (3)	S—C13—C14—C15	−176.00 (19)
C10—C5—C6—C7	−177.4 (2)	C13—C14—C15—C16	1.4 (4)
C4—C5—C6—C11	−179.2 (2)	C14—C15—C16—C17	−0.1 (4)
C10—C5—C6—C11	2.0 (3)	C14—C15—C16—Cl	−179.48 (19)
C5—C6—C7—O1	178.34 (19)	C15—C16—C17—C18	−0.8 (4)
C11—C6—C7—O1	−1.1 (3)	Cl—C16—C17—C18	178.60 (19)
C5—C6—C7—C2	−1.1 (3)	C16—C17—C18—C13	0.3 (4)
C11—C6—C7—C2	179.4 (2)	C14—C13—C18—C17	1.0 (3)
C8—O1—C7—C6	−179.4 (2)	S—C13—C18—C17	175.08 (18)

### Hydrogen-bond geometry (Å, °)

**Table:**

Cg1 and Cg2 are the centroids of the C13–C18 4-chlorophenyl ring and the C2–C7 benzene ring, respectively.

**Table:**

*D*—H···*A*	*D*—H	H···*A*	*D*···*A*	*D*—H···*A*
C11—H11A···Cg1^i^	0.96	2.76	3.613 (3)	148
C12—H12B···Cg2^ii^	0.96	2.83	3.653 (3)	144

Symmetry codes: (i) *x*−1/2, −*y*+3/2, *z*; (ii) *x*, *y*, *z*−1.
